# Ovariotomy

**Published:** 1869-08-15

**Authors:** 


***Ovariotomy.***

Since his last report (June, 1868), M. Kœberle has performed this operation twenty-five times (up to April 1st, 1869), and tabulates them thus:

| | | | |
|---|---|---|---|
| Without adhesions, | 8 cases; | 8 cures. | |
| With slight adhesions, | 10 " | 10 " | |
| With important adhesions, | 7 " | 2 " | 5 deaths. |
| Total, . . . | 25 cases; | 20 cures; | 5 deaths. |

Among these twenty-five new cases there were six patients aged from 48 to 65 years. Of these, one aged 52 years died. There were three cases of double ovariotomy. These three cases were followed by recovery.—*Ibid.*

